# Phenotypes of Older Adults Planning for Alzheimer's Disease Support

**DOI:** 10.1111/jgs.70401

**Published:** 2026-03-20

**Authors:** Amber P. Miller‐Winder, Raven R. Relerford, Allison Schierer, Alaine Murawski, Charles Olvera, Laura M. Curtis, Kwang‐Youn Kim, Vanessa Ramirez‐Zohfeld, Lee A. Lindquist

**Affiliations:** ^1^ Division of Geriatrics, Feinberg School of Medicine Northwestern University Chicago Illinois USA; ^2^ Division of General Internal Medicine, Feinberg School of Medicine Northwestern University Chicago Illinois USA; ^3^ Department of Preventive Medicine, Feinberg School of Medicine Northwestern University Chicago Illinois USA

**Keywords:** Alzheimer's disease, caregivers, long term care supports, older adults, qualitative anaylsis

## 1 Background

Older adults living with Alzheimer's disease (AD) require progressively greater levels of support (e.g., in‐home assistance and supportive facilities) [1, 2]. Yet, older adults and their families vary widely in how they perceive and plan for future support needs [3]. Recognizing these diverse attitudes toward long‐term care (LTC) planning, we aimed to characterize and model the distinct phenotypes that emerge as older adults consider their future AD‐related support needs.

## 2 Methods

The Plan Your Lifespan study examines older adult decision‐making processes about support, in the event of developing AD [4]. Subjects were recruited from the LitCog study; a cohort of older adults age 65+ who participate in extensive neuropsychological cognitive testing every 2.5 years [5]. Subjects were provided PlanYourLifespan.org, which facilitates aging‐in‐place and LTC decision‐making [6, 7, 8, 9]. Subjects were interviewed every 6 months for 42‐months, about cognitive, social, functional, environmental factors, and hypothetical scenarios such as if they were to develop AD, have they decided on support preferences. All interviews from baseline‐42 months were analyzed.

We employed mixed analytic methods through qualitative coding and generalized linear mixed modeling (GLMM). Mixed‐methods analysis included analyzing open‐ended, qualitative responses about perceptions of planning in the event of developing AD. The responses were coded using constant comparative analysis with triangulation of themes. GLMM was used to identify factors associated with statistically significant increases or decreases in the likelihood of planning for AD.

## 3 Results

**FIGURE 1:**
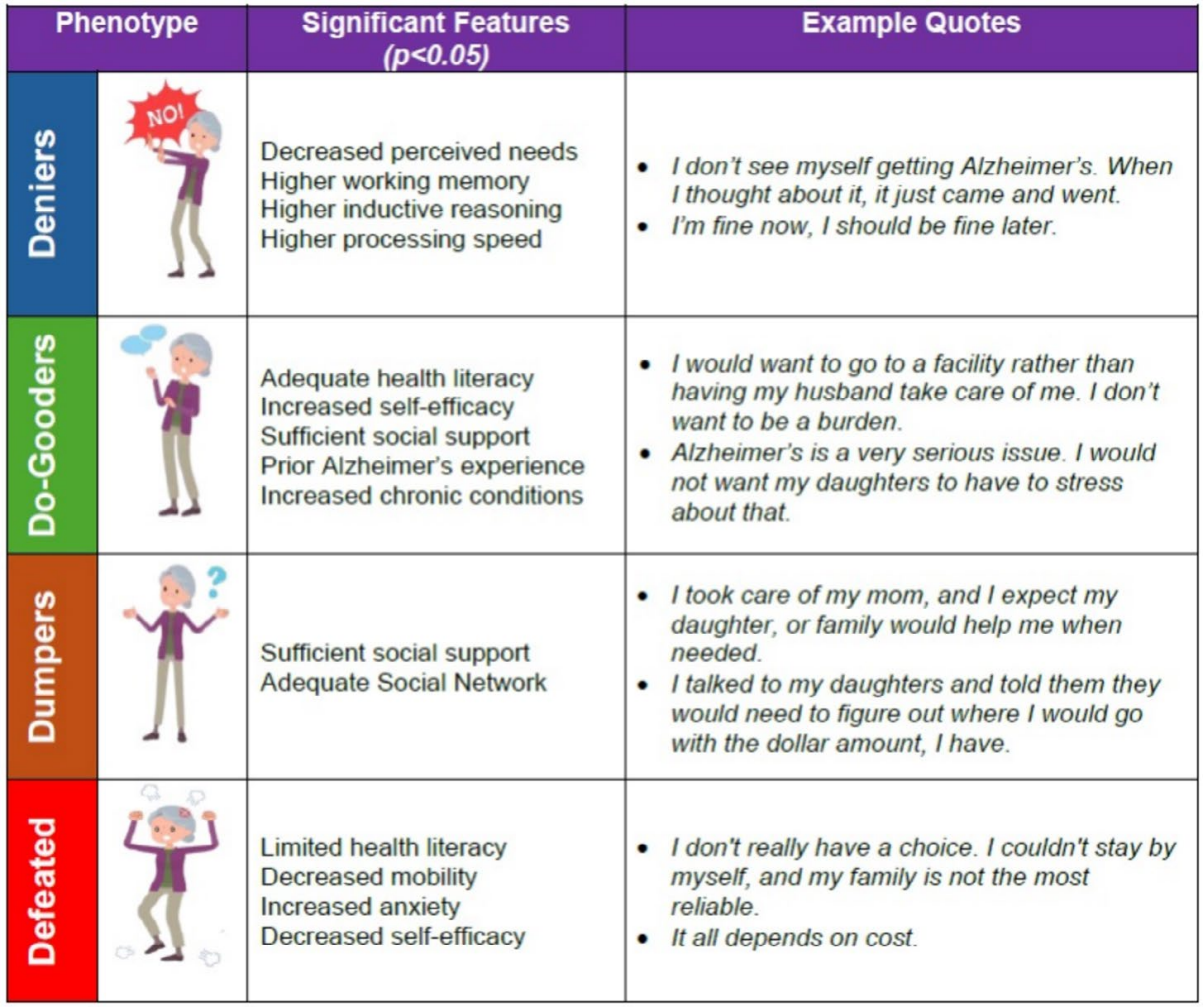
Phenotypes of older adults in planning for future long term support needs.

Of the 293 subjects enrolled (mean age = 73.5 years, 72.7% female, 40.4% under‐represented minority, 40.6% limited health literacy), almost half (47.4%, *n* = 139) self‐reported worsening memory loss at 18 months post‐baseline, with 42.4% (*n* = 59) experiencing memory loss episodes weekly and 18% (*n* = 25) monthly (Table 1). Neuropsychological testing revealed 22.2% (*n* = 65) had mild or moderate cognitive impairment. Nearly half of those subjects (46.2%, *n* = 30) denied experiencing any cognitive loss. Of subjects testing at normal cognitive levels, 47.8% (*n* = 104) reported new or worsening memory loss. At baseline, 15% (*n* = 44) had never considered the possibility of developing AD in the future. Qualitative findings revealed four planning phenotypes: (a) Deniers, (b) Do‐gooders, (c) Dumpers, and (d) Defeated (Figure 1).

**TABLE 1:** Baseline demographics (*n* = 293).

Characteristics	Percent (*n*)
Age, mean (SD)	73.8 (5.23)
Sex, female	72.7 (213)
Race (total may exceed 100%)	
White	65.2 (191)
Black	28.3 (83)
Asian	2.7 (8)
Other	2.7 (8)
Ethnicity—Hispanic	4.4 (13)
Education, %	
High school or less	14.3 (42)
Some college	18.4 (54)
Bachelor's degree or higher	66.6 (195)
Income	
< $20,000	13.0 (38)
$20,000–$34,999	16.7 (49)
$35,000–$49,999	11.6 (34)
> $50,000	54.3 (159)
Retired/Unemployed	71.7 (210)
Married	47.1 (138)

*Deniers* rejected the need to plan for future health events, such as AD. They displayed decreased perceived support needs (OR 0.357; *p* < 0.05), higher working memory (OR 0.88; *p* < 0.05), higher inductive reasoning (OR 0.90; *p* < 0.05), higher processing speed (OR 0.95; *p* < 0.05), and higher busyness (OR 0.95; *p* < 0.05).

*Do‐gooders* created LTC plans to avoid burdening others. Do‐gooders exhibited adequate health literacy (OR 5.06; *p* < 0.05), increased self‐efficacy (OR 1.07; *p* < 0.01), and more cognitive impairment (OR 1.46; *p* < 0.05). They reported sufficient social support (OR 2.82; *p* < 0.05), prior experiences with others with AD or memory loss (OR 1.90; *p* < 0.05), completion of a living will (OR 2.43; *p* < 0.05), and had a higher number of chronic conditions (OR 1.20; *p* < 0.05).

*Dumpers* left their future health care planning and assist with support needs to others. This dumping was possible since they had sufficient social support (OR 3.39; *p* < 0.05) and adequate social networks (OR 1.08; *p* < 0.05).

*Defeated* subjects did not plan due to perceived internal (e.g., too sick) or external limitations (e.g., lack of savings). They exhibited below average health activation scores (OR 0.65; *p* < 0.05), limited health literacy (OR 1.84; *p* < 0.05), decreased physical function (OR 0.95; *p* < 0.05), increased anxiety scores (OR 1.04; *p* < 0.05), and decreased self‐efficacy (OR 0.96; *p* < 0.05).

## 4 Discussion

When planning for future LTC support needs, four distinct phenotypes for older adults were identified. *Deniers* believed that they would never require future support and avoided future‐oriented thinking. Clinicians might be able to intercede in realistically pointing out their prognosis and future needs. *Do‐gooders* were highly motivated to avoid burdening others. With prior experiences with others who had AD, *do‐gooders* may have a greater understanding of the effect AD has on the family. *Do‐gooders* commonly tested at levels of cognitive decline, and their awareness of cognitive decline and prior experience with others with AD could explain the urgency of creating LTC plans [9]. *Dumpers* had sufficient social support and social networks on which they could depend. Prior research has shown that while some older adults anticipate their assistance, families may not be aware of these designs [10]. Therefore, providers can help encourage communication between families and older adults. *Defeated* subjects had decreased self‐efficacy, increased anxiety, and often unreliable social networks. They were overwhelmed and unable to form plans. Social work might be tapped to intervene with these older adults. Across all phenotypes, planning behaviors were dependent on health literacy levels, presence and availability of social support, and personal experience with others who had AD. Future research will reveal how these phenotypes evolve over time, with changing social networks, personal experiences, and health needs. Identifying these phenotypes and trajectories will be essential for designing interventions that assist older adults at risk for AD in making timely and informed long‐term care decisions.

## Author Contributions

All authors met criteria for authorship by (1) conception and design of the study: L.A.L., V.R.‐Z., (2) data acquisition: APM, AS, AM, R.R.R., V.R.‐Z., (3) analysis and interpretation of data: All authors. (4) manuscript drafting: All authors. (5) revising the manuscript critically for important intellectual content: All authors. (6) approval of the version of the manuscript to be published: All authors.

## Funding

This research is supported through grants from the NIH/NIA (R01AG058777, R01AG30611, and P30AG059988).

## Disclosure

All statements in this manuscript, including its findings and conclusions, are solely those of the authors.

## Conflicts of Interest

The authors declare no conflicts of interest.

## References

[1] K. Lam , I. Cenzer , and K. E. Covinsky , “Return to Community Living and Mortality After Moving to a Long‐Term Care Facility: A Nationally Representative Cohort Study,” Journal of the American Geriatrics Society 71, no. 2 (2023): 569–576. 36420717 10.1111/jgs.18144 PMC9957796

[2] K. Lam , I. Cenzer , C. R. Levy , D. D. Matlock , A. K. Smith , and K. E. Covinsky , “The Natural History of Disability and Caregiving Before and After Long‐Term Care Entry,” JAMA Internal Medicine 183, no. 12 (2023): 1295–1303. 37930717 10.1001/jamainternmed.2023.5427 PMC10628843

[3] I. Cohen , R. Relerford , C. Olvera , V. Ramirez‐Zohfeld , A. Miller‐Winder , and L. A. Lindquist , “What Changed Your Mind? Influencers of Older Adults Changing Decisions About Aging‐In‐Place Versus Long‐Term Care,” Journal of the American Geriatrics Society 73, no. 8 (2025): 2512–2516. 40214161 10.1111/jgs.19475 PMC12353053

[4] A. Schierer , A. Miller‐Winder , A. Murawski , et al., “Fluctuating Decision Making About Aging‐In‐Place/Long Term Care Planning Among Older Adults,” Journal of the American Geriatrics Society 71, no. 12 (2023): 3938–3940. 37555587 10.1111/jgs.18536 PMC10840837

[5] L. A. Lindquist , V. Ramirez‐Zohfeld , P. Sunkara , et al., “Advanced Life Events (ALEs) That Impede Aging‐In‐Place Among Seniors,” Archives of Gerontology and Geriatrics 64 (2016): 90–95. 26952382 10.1016/j.archger.2016.01.004 PMC6065259

[6] L. A. Lindquist , V. Ramirez‐Zohfeld , C. Forcucci , P. Sunkara , and K. A. Cameron , “Overcoming Reluctance to Accept Home‐Based Support From an Older Adult Perspective,” Journal of the American Geriatrics Society 66, no. 9 (2018): 1796–1799. 30155882 10.1111/jgs.15526

[7] L. A. Lindquist , R. Muhammad , A. P. Miller‐Winder , et al., “Rationale and Study Design for Decision Making & Implementation of Aging‐In‐Place/Long Term Care Plans Among Older Adults,” Contemporary Clinical Trials Communications 22 (2021): 100756. 33869887 10.1016/j.conctc.2021.100756 PMC8040099

[8] L. A. Lindquist , V. Ramirez‐Zohfeld , P. D. Sunkara , et al., “PlanYourLifeSpan.org—An Intervention to Help Seniors Make Choices for Their Fourth Quarter of Life: Results From the Randomized Clinical Trial,” Patient Education and Counseling 100, no. 11 (2017): 1996–2004. 28689855 10.1016/j.pec.2017.06.028 PMC6065258

[9] L. A. Lindquist , A. P. Miller‐Winder , A. Schierer , et al., “Aspects of Cognition That Impact Aging‐In‐Place and Long‐Term Care Planning,” Journal of the American Geriatrics Society 70 (2022): 2646–2652. 35726136 10.1111/jgs.17927 PMC9489627

[10] A. P. Miller‐Winder , A. Schierer , R. R. Relerford , et al., “Subjective Cognitive Decline and Missed Aging‐In‐Place/Long‐Term Care Planning,” Journal of the American Geriatrics Society 71, no. 10 (2023): 3314–3316. 37235515 10.1111/jgs.18425 PMC10592648

